# Persistent pulmonary hypertension of the newborn

**DOI:** 10.1186/s40748-015-0015-4

**Published:** 2015-06-03

**Authors:** Vinay Sharma, Sara Berkelhamer, Satyan Lakshminrusimha

**Affiliations:** Department of Pediatrics (Neonatology), Hennepin County Medical Center, 701 Park Avenue, Shapiro Building, Minneapolis, MN 55415 USA; Department of Pediatrics, Women and Children’s Hospital of Buffalo, 219 Bryant St, Buffalo, New York 14222 USA

**Keywords:** Nitric Oxide, Fetal circulation, Pulmonary vascular remodeling, Meconium aspiration, Surfactant, Natriuretic peptide, Meconium aspiration, Prostaglandin E1, Sildenafil, Milrinone

## Abstract

Persistent pulmonary hypertension of the newborn (PPHN) is characterized by elevated pulmonary vascular resistance resulting in right-to-left shunting of blood and hypoxemia. PPHN is often secondary to parenchymal lung disease (such as meconium aspiration syndrome, pneumonia or respiratory distress syndrome) or lung hypoplasia (with congenital diaphragmatic hernia or oligohydramnios) but can also be idiopathic. The diagnosis of PPHN is based on clinical evidence of labile hypoxemia often associated with differential cyanosis. The diagnosis is confirmed by the echocardiographic demonstration of – (a) right-to-left or bidirectional shunt at the ductus or foramen ovale and/or, (b) flattening or leftward deviation of the interventricular septum and/or, (c) tricuspid regurgitation, and finally (d) absence of structural heart disease. Management strategies include optimal oxygenation, avoiding respiratory and metabolic acidosis, blood pressure stabilization, sedation and pulmonary vasodilator therapy. Failure of these measures would lead to consideration of extracorporeal membrane oxygenation (ECMO); however decreased need for this rescue therapy has been documented with advances in medical management. While trends also note improved survival, long-term neurodevelopmental disabilities such as deafness and learning disabilities remain a concern in many infants with severe PPHN.

**Funded by:** 1R01HD072929-0 (SL)

## Introduction

Persistent pulmonary hypertension of newborn (PPHN) is secondary to failure of normal circulatory transition at birth. It is a syndrome characterized by elevated pulmonary vascular resistance (PVR) that causes labile hypoxemia due to decreased pulmonary blood flow and right-to-left shunting of blood. Prompt diagnosis and management, including a timely referral to a tertiary care center can dramatically improve outcome. Its incidence has been reported as 1.9 per 1000 live births (0.4–6.8/1000 live births) with mortality rate ranging between 4–33% [1]. This syndrome complicates the course of about 10% of term and preterm infants with respiratory failure and remains a source of considerable morbidity and mortality. This review includes a discussion of fetal circulation and circulatory transition at birth, as well as the pathophysiology, diagnosis, management and long term complications of PPHN.

## Findings

### Fetal circulation

Pulmonary hypertension with reduced pulmonary blood flow is a normal physiologic state in the fetus. This is because the placenta, not the lung, serves as the organ of gas exchange. Most of the right ventricular output crosses the ductus arteriosus to the aorta (approximately 8-10% of combined ventricular output in an ovine fetus and 13% -21% in human fetuses) [2-4]. Even though pulmonary vascular cross-sectional area increases with fetal lung growth, PVR increases with gestational age when corrected for lung or body weight, suggesting that pulmonary vascular tone increases during late gestation. Multiple pathways appear to be involved in maintaining high pulmonary vascular tone prior to birth. Apart from mechanical factors (fluid-filled lung), hypoxic pulmonary vasoconstriction and circulating vasoconstrictors such as endothelin-1 and products of the prostaglandin pathway (i.e. leukotriene and thromboxane) play a significant role in maintaining high fetal PVR [5].

Serotonin increases fetal PVR [6,7]. The use of selective serotonin reuptake inhibitors (SSRIs) during the last half of pregnancy has been associated with an increased incidence of PPHN in at least three human population studies [8-11]. While the mechanism by which SSRIs induce pulmonary hypertension in newborn is not known, it is speculated that higher drug-induced serotonin levels result in pulmonary vasoconstriction. However, recent studies have questioned the association between maternal SSRI intake and PPHN [12,13]. Furthermore, the severity of PPHN has not been well described and a recent report observed no differences in right pulmonary artery Doppler pulsatility index (PI) in fetuses of mothers exposed to SSRI antidepressants [14]. At present, maternal physical and psychological well-being should be the primary factor guiding anti-depressant therapy during pregnancy and postpartum period.

**Figure 1:**
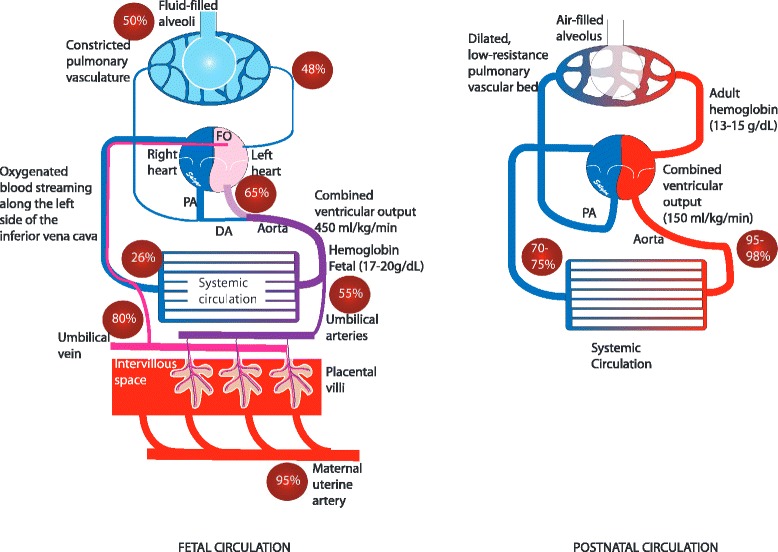
Fetal and postnatal (adult) circulation: in fetuses, placenta is the organ of gas exchange. The umbilical vein carries oxygenated blood from the placenta has the highest fetal oxygen saturation of approximately 80%. Oxygen saturation in various blood vessels is shown in crimson circles. Oxygenated blood is streamed through the ductus venosus and along the left margin of the inferior vena cava. In the heart, oxygenated blood gets shunted across the foramen ovale (FO) to the left heart to supply the cerebral and coronary circulation. Poorly oxygenated blood from the upper half of the body goes to the right side of the heart, the main pulmonary artery and gets shunted through the ductus arteriosus (DA) to the descending aorta. Blood in the descending aorta has a saturation of around 55 %, and this blood flows back to placenta for oxygenation through umbilical arteries. In spite of relative hypoxia, adequate oxygen delivery to the fetal tissues is maintained by (a) high cardiac output, (b) high hemoglobin level in the term fetus and (c) the presence of fetal hemoglobin (HbF) with high oxygen affinity. The saturation gradient across the placenta (85 – 55 = 30%) is similar to the saturation gradient across the lungs in adults (98 – 70% = 28%) but is achieved at a much lower partial pressure of oxygen (PO_2_ ~ 32 mmHg in the umbilical vein) in the fetus compared to the adult (PaO_2_ ~ 95 mmHg) limiting the fetal risk for oxygen toxicity. *(copyright Satyan Lakshminrusimha)*.

The fetus is in a physiological state of hypoxemia (relative to postnatal standards) with PaO_2_ of around 25 mmHg, a level that promotes normal growth and differentiation of pulmonary vascular cells and supports normal branching morphogenesis [15]. However, a normal fetus is not hypoxic and adequate oxygen delivery to the fetal tissues is maintained by multiple mechanisms including high cardiac output *in utero*, high hemoglobin level in the term fetus, and presence of fetal hemoglobin (HbF) with high oxygen affinity (Figure 1).

### Transitional circulation

A series of circulatory events take place at birth to ensure a smooth transition from fetal to extra-uterine life. Clamping of the umbilical cord removes the low resistance placental circulation, increasing systemic arterial pressure. Simultaneously, various mechanisms operate to rapidly reduce pulmonary arterial pressure and increase pulmonary blood flow. The most important stimulus to promote pulmonary vasodilation appears to be ventilation of the lungs and an increase in oxygen tension [5]. There is improved oxygenation of the pulmonary vascular bed, further decreasing PVR [16]. An eight-fold increase in pulmonary blood flow occurs, which raises left atrial pressure, closing the foramen ovale. As PVR drops lower than systemic vascular resistance (SVR), there is a reversal of flow across the ductus arteriosus (from aorta to pulmonary artery or left-to-right). The increase in arterial oxygen saturation leads to closure of the ductus arteriosus and ductus venosus. In the final phase of neonatal pulmonary vascular transition, a further decline in PVR is accompanied by rapid structural remodeling of the entire pulmonary bed, from the main pulmonary arteries to the capillaries [17].

### Mediators of circulatory transition at birth

**Figure 2:**
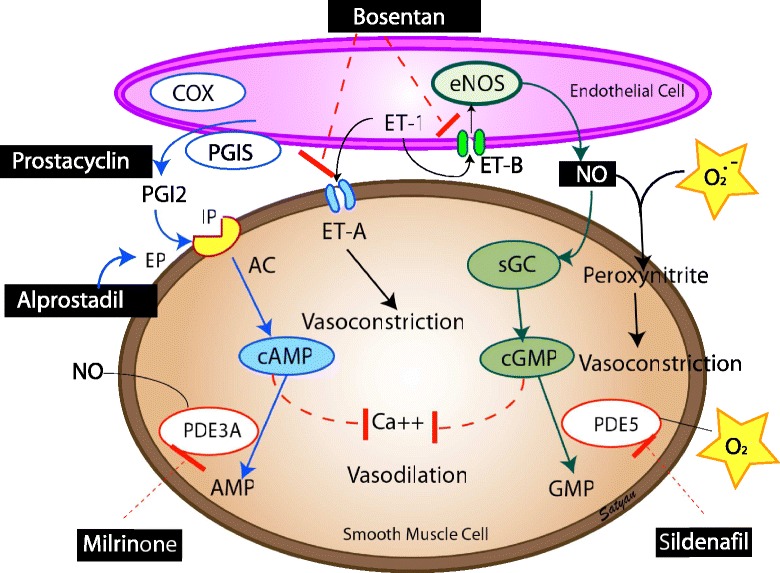
Endothelium derived vasodilators – prostacyclin (PGI_2_) and nitric oxide (NO) and vasoconstrictor (endothelin, ET-1). Cyclooxygenase (COX) and prostacyclin synthase (PGIS) are involved in the production of prostacyclin. Prostacyclin acts on its receptor (IP) in the smooth muscle cell and stimulates adenylate cyclase (AC) to produce cyclic adenosine monophosphate (cAMP). Cyclic AMP is broken down by phosphodiesterase 3A (PDE 3A, the enzyme most prevalent in vasculature) in the smooth muscle cell. Milrinone inhibits PDE 3A and increases cAMP levels in arterial smooth muscle cells and cardiac myocytes resulting in pulmonary (and systemic) vasodilation and inotropy. Nitric oxide (NO) stimulates PDE 3A [151,152]. Endothelin is a powerful vasoconstrictor and acts on ET-A receptors in the smooth muscle cell and increases ionic calcium concentration. A second endothelin receptor (ET-B) on the endothelial cell stimulates NO release and vasodilation. Endothelial nitric oxide synthase (eNOS) produces NO which diffuses from the endothelium to the smooth muscle cell and stimulates soluble guanylate cyclase (sGC) enzyme to produce cyclic guanosine monophosphate (cGMP). Cyclic GMP is broken down by PDE5 enzyme in the smooth muscle cell. Sildenafil inhibits PDE5 and increases cGMP levels in pulmonary arterial smooth muscle cells. Natriuretic peptides stimulate particulate guanylate cyclase (pGC) to produce cGMP. Cyclic AMP and cGMP reduce cytosolic ionic calcium concentrations and induce smooth muscle cell relaxation and pulmonary vasodilation. Nitric oxide is a free radical and can avidly combine with superoxide anions to form a toxic vasoconstrictor, peroxynitrite. Hence, the bioavailability of NO in a tissue is determined by the local concentration of superoxide anions. Hyperoxic ventilation with 100% oxygen can increase the risk of formation of superoxide anions in the pulmonary arterial smooth muscle cells and limit the bioavailability of NO and stimulate PDE5 activity [81]. Medications used in PPHN are shown in black boxes *(copyright Satyan Lakshminrusimha)*.

Vascular endothelium releases several vasoactive products that play a primary role in pulmonary transition at birth. Pulmonary endothelial nitric oxide (NO) production increases markedly at the time of birth. Oxygen is believed to be an important catalyst for this increased NO production, although the precise mechanism is not clear. It increases oxidative phosphorylation and the release of red blood cell ATP which is a pulmonary vasodilator during fetal life and a potential stimulus for endothelial NO production [18,19]. In intrapulmonary arteries isolated from near-term fetal sheep, both basal and stimulated NO release increase with escalating oxygen tension [20]. The shear stress resulting from increased pulmonary blood flow and increased oxygenation also induce endothelial nitric oxide synthase (eNOS) expression, thus contributing to NO-mediated pulmonary vasodilation after birth [5]. Nitric oxide exerts its action through soluble guanylate cyclase (sGC) and cGMP (Figure 2). The importance of the NO-cGMP pathway in facilitating normal transition has been demonstrated by acute or chronic inhibition of nitric oxide synthase (NOS) in fetal lambs, which produces pulmonary hypertension following delivery [21,22]. Data indicate that eNOS dysfunction induced through increased levels of asymmetric dimethyl arginine (ADMA), a competitive endogenous inhibitor of NOS [23], or by decreased synthesis of the NOS substrate L-arginine [24], may result in pulmonary vasoconstriction.

The arachidonic acid-prostacyclin pathway also plays an important role in the transition at birth. The cyclooxygenase enzyme acts on arachidonic acid to produce prostaglandin endoperoxides. Prostaglandins activate adenylate cyclase to increase cAMP concentrations in vascular smooth muscle cells (Figure 2). Cyclooxygenase-1 in particular is upregulated during late gestation, leading to an increase in prostacyclin production in the third trimester and early postnatal life [25,26]. Inhibition of prostacyclin production by non-steroidal anti-inflammatory drugs (NSAIDs) during late pregnancy has been associated with PPHN although this association has been recently called into question [27].

Finally, atrial natriuretic peptide (ANP), B-type natriuretic peptide (BNP) and C-type natriuretic peptide (CNP) dilate fetal pulmonary vasculature by increasing cGMP through particulate guanylate cyclase (pGC) [28] and may play a role in pulmonary vascular transition at birth (Figure 2).

### Pathophysiology of PPHN

**Figure 3:**
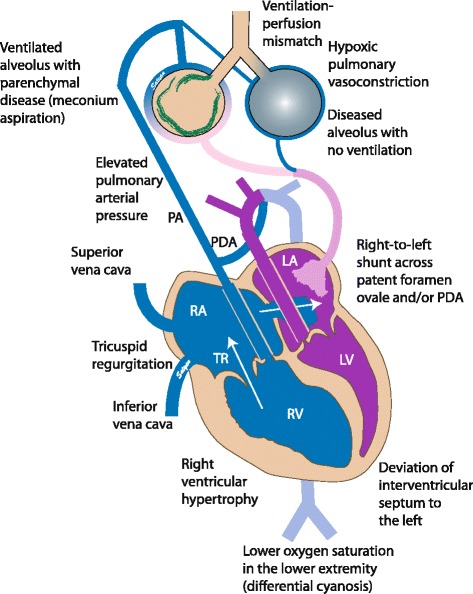
Pathophysiology of PPHN. Parenchymal lung disease and ventilation-perfusion (V/Q) mismatch result in hypoxemia. Increased pulmonary vascular resistance results in reduced pulmona ry blood flow and right to left shunt through PDA and/or PFO. Pulmonary hypertension is often associated with systemic hypotension with deviation of the interventricular septum to the left. The right subclavian artery (and blood flowing to the right upper extremity) is always preductal. The left subclavian artery may be preductal, juxtaductal or postductal. Hence, preductal oxygen saturations should be obtained from the right upper extremity and compared with lower extremity to assess differential cyanosis. PA – pulmonary artery; RV – right ventricle; LV – left ventricle; TR – tricuspid regurgitation; RA – right atrium; LA – left atrium; PDA – patent ductus arteriosus; PFO – patent foramen ovale. *(copyright Satyan Lakshminrusimha)*.

Disruption of normal neonatal circulatory transition results in failure to resolve fetal pulmonary hypertension and leads to the persistence of fetal pulmonary hypertension or PPHN. High pulmonary pressure decreases the blood flow to the lungs. Ventilation-perfusion (VQ) mismatch (Figure 3) and extrapulmonary right-to-left shunting of deoxygenated blood across the patent foramen ovale (PFO) and patent ductus arteriosus (PDA) result in cyanosis. **Differential cyanosis** (saturation in the lower limb is 5-10% lower than right upper limb) occurs due to pulmonary artery to aorta shunt through the PDA (Figure 3). If the PDA is closed and the shunt exclusively is at the PFO level, the degree of cyanosis is similar in both upper and lower limbs. **Labile hypoxemia** (marked change in oxygen saturation with minimal or no change in ventilator settings) is characteristic of PPHN and is due to change in the volume of right-to-left shunt secondary to subtle changes in the delicate balance between PVR and SVR.

### Classification

PPHN can be characterized as one of four types:

1. *Maladaptation*: Secondary to lung parenchymal diseases such as meconium aspiration syndrome (MAS), respiratory distress syndrome (RDS), or pneumonia
2. *Maldevelopment*: Lung with normal parenchyma and remodeled pulmonary vasculature, also known as idiopathic PPHN
3. *Underdevelopment*: Hypoplastic vasculature as seen in CDH and other causes of pulmonary hypoplasia (oligohydramnios secondary to Potter’s Syndrome, renal disease or chronic leakage of amniotic fluid)
4. *Intrinsic obstruction*: high viscosity due to polycythemia resulting in intravascular obstruction and elevated PVR

#### Maladaption/Parenchymal lung diseases resulting in secondary PPHN

a) *Meconium aspiration syndrome* (MAS) in newborns leads to acute respiratory failure with a mortality of up to 10% [29]. Meconium causes chemical pneumonitis and surfactant inactivation that leads to ventilation-perfusion mismatch. Resulting hypoxemia and hypercarbia cause pulmonary vasoconstriction and PPHN. Structural studies of the lung at postmortem in fatal cases of meconium aspiration suggest that antecedent pulmonary vascular abnormalities exacerbated the postnatal pulmonary hypertension [30]. Decreased eNOS expression has also been reported in umbilical venous endothelial cell cultures from human infants with meconium staining who develop PPHN [31]. Meconium components incite an inflammatory response with release of cytokines and increase the production of vasoconstrictors including endothelin and thromboxane [32]. The incidence of MAS has decreased in developed countries but continues to be prevalent in resource-limited settings often associated with asphyxia [33].

Management of a neonate born through meconium stained amniotic fluid has changed dramatically over the last decade. Amnioinfusion, suctioning at the perineum and tracheal suctioning in vigorous infants did not alter the incidence of MAS in multicenter randomized trials [34-36]. The current guidelines recommend tracheal suctioning only if the infant born through meconium stained amniotic fluid is not vigorous at birth [37]. Recent data from a randomized trial and a translational study have pushed further to question the benefit of tracheal suctioning of meconium at birth even if the newborn is not vigorous [38,39]. Additional studies are required to evaluate the effect of tracheal suctioning in MAS and the incidence and severity of PPHN.

*b) Pneumonia and sepsis* often present with elevated PVR associated with systemic hypotension and decreased SVR. In addition, some infants with sepsis have myocardial dysfunction resulting in pulmonary venous hypertension due to elevated left atrial pressures [40].

*c) Pulmonary hypertension in premature infants:* Although PPHN is traditionally considered a disease of term and late preterm infants, it is increasingly being diagnosed in extremely preterm infants [41]. Some preterm infants with RDS present with PPHN in the first few days of life [42] while preterm infants with bronchopulmonary dysplasia (BPD) may be diagnosed with pulmonary hypertension later in the hospital course or after discharge from the Neonatal Intensive Care Unit (NICU). Preterm infants with fetal growth restriction and born after prolonged rupture of membranes are at higher risk for developing pulmonary hypertension [43]. Pulmonary vascular disease significantly increases morbidity and mortality in BPD [44].

#### Maldevelopment/Idiopathic PPHN (“Black-lung PPHN)

Some cases of PPHN are not secondary to parenchymal lung disease and are referred to as idiopathic or “black-lung” (referring to paucity of pulmonary vascularity and absence of lung disease) PPHN. Idiopathic pulmonary hypertension is secondary to remodeled pulmonary arteries, characterized by smooth muscle hyperplasia and extension of smooth muscle in intra-acinar arteries. The abnormal structural remodeling of the pulmonary circulation as seen in PPHN affects the responsiveness to vasodilator stimuli, and may prevent the access of NO to the vascular smooth muscle cells [45]. Maternal use of NSAIDs during third trimester of pregnancy can lead to premature closure of ductus arteriosus [46] and “black-lung” PPHN. Ovine fetal ligation or constriction of ductus arteriosus can produce similar vascular changes and is widely used to replicate PPHN in newborn lambs, showing the same pulmonary vascular findings as idiopathic pulmonary hypertension [47].

#### Underdevelopment / Pulmonary hypoplasia

Congenital diaphragmatic hernia (CDH) is developmental defect in the diaphragm separating the thorax and the abdomen and is the most important cause of pulmonary hypoplasia resulting in PPHN. This defect leads to a herniation of the abdominal viscera into the thoracic cavity. CDH occurs in 1/2,500 to 5,000 live births. CDH has a mortality rate of 20-30% and the degree of associated pulmonary hypoplasia and the severity of pulmonary hypertension remain the major determinants of survival [48]. Pulmonary vascular abnormalities in CDH include a decreased number of pulmonary arteries per unit lung volume and peripheral muscularization of small arteries, with medial and adventitial thickening [49]. In spite of marked improvement in survival of PPHN resulting from other causes, the mortality and need for ECMO remain high in infants with CDH.

Pulmonary hypoplasia secondary to renal dysfunction and oligohydramnios or thoracic dystrophy can be associated with pulmonary hypertension [50-52]. Prolonged rupture of membranes is also a risk factor for pulmonary hypertension in preterm infants [53].

### Alveolar capillary dysplasia (ACD)

Alveolar capillary dysplasia is generally associated with malalignment of the pulmonary veins (ACD/MPV) and produces respiratory failure early in life and carries a mortality rate that approaches 100% [54]. Recent reports of infants presenting with fulminant symptoms of ACD/MPV well beyond the neonatal period, even as late as 7 months of age, have begun to emerge, challenging the established phenotype and offering the possibility that long-term survivors with milder forms of the disease may exist. [55] FOXF1 transcription factor gene or deletions upstream to FOXF1 were identified in 40% of cases [56]. Blood testing to screen for these defects is now available, but a negative result does not preclude the diagnosis, and histological examination of lung tissue remains the gold standard for diagnosis. A lung biopsy to rule out ACD should be considered for neonates who do not respond to conventional medical management or fail attempts at ECMO decannulation.

### Diagnosis

In a term or near-term infant with respiratory distress, the initial evaluation should include simultaneous measurement of pre- and post-ductal oxygen saturation, a chest X-ray and an arterial blood gas. Hypoxemia disproportionate to the severity of parenchymal disease on a chest radiograph should suggest idiopathic PPHN (or cyanotic heart disease). Evidence of the underlying parenchymal disease such as RDS, MAS, or pneumonia may be seen on chest X-ray in secondary PPHN. A complete blood count with differential is often obtained on admission to evaluate for high hematocrit level (polycythemia and increased viscosity contributing to intrinsic vascular obstruction) and to evaluate the risk of underlying infection.

Differentiating PPHN from cyanotic CHD soon after admission is of paramount importance. Preductal and postductal oxygen saturation/PaO_2_ measurements are used to differentiate PPHN from structural heart disease. Saturation differences of > 5-10% or PaO_2_ differences of 10–20 mmHg between right upper limb and lower limbs are considered significant. In neonates with PPHN and atrial-level right-to-left shunting without a significant ductal shunt, both the right arm and the right leg saturations will be low. Conversely, babies with PDA and coarctation of the aorta might have differential cyanosis. In PPHN, hypoxemia is often labile unlike fixed hypoxemia seen in cyanotic CHD. Hyperoxia test (obtaining an arterial gas after 15 minutes of exposure to 100% oxygen) and/or hyperoxia-hyperventilation (hyperoxia and alkalosis to induce pulmonary vasodilation and improve PaO_2_) is no longer widely practiced due to the known adverse effects of hyperoxia and alkalosis. It can be avoided by confirming elevated pulmonary pressures by an early echocardiogram, when available.

Echocardiography is gold standard to confirm the diagnosis, and to monitor the efficacy of specific therapeutic interventions [57]. Measurement of the direction of ductal and foramen ovale shunt, flattening or left-deviation of the interventricular septum and tricuspid regurgitation velocity with simultaneous systemic blood pressure measurement provides an indication of right-sided pressures and hemodynamic physiology.

B-type natriuretic peptide (BNP) concentrations in plasma correspond well with echocardiographic findings of ventricular strain [58]. Reynolds et al. suggested BNP as an early indicator of PPHN in the presence of respiratory illness in neonates without CHD [59]. BNP has been proposed as a biomarker in PPHN, especially to assess efficacy of treatment and to predict rebound PPHN [59,60]. However, its value in the practical management of PPHN is presently unclear. Some centers obtain serial (monthly) echocardiograms with BNP levels to screen for pulmonary hypertension associated with BPD in preterm infants.

### Severity of PPHN

Severity of PPHN is commonly assessed by oxygenation index (OI) and Alveolar-arterial oxygen difference (AaDO_2_). OI is more commonly used during medical management of PPHN since it takes ventilator support into the consideration and is calculated as

$$ \mathrm{O}\mathrm{I}\kern0.5em =\kern0.5em \mathrm{MAP}\kern0.5em \times \kern0.5em {\mathrm{FiO}}_2\kern0.5em \times \kern0.5em 100/{\mathrm{PaO}}_2 $$

where MAP is the mean airway pressure in cmH_2_O, FiO_2_ is the fraction of inspired oxygen, and PaO_2_ is partial pressure of oxygen in arterial blood (in mmHg). Hypoxemic respiratory failure can be classified into mild (OI ≤ 15), moderate (OI > 15 to 25), severe (OI 25 to 40) and very severe (OI > 40) [61]. Disadvantages of OI include:(a) it can be manipulated by changing FiO_2_ or MAP or based on the type of ventilator; (b) it requires arterial access; (c) the value may vary based on the site of arterial access – right radial (preductal) vs. umbilical or posterior tibial (postductal). More recently, oxygen saturation index (OSI = MAP × FiO_2_ × 100/Preductal SpO_2_) has been used in patients without arterial access [62]. If preductal SpO_2_ is in the 70-99% range, OSI corresponds to approximately half of OI (OSI of 8 = OI of 16) [63]. More research evaluating the clinical role for this non-invasive index is needed prior to its widespread use.

AaDO_2_ is the difference between Alveolar partial pressure of oxygen and arterial partial pressure of oxygen and is calculated using the following formula.

$$ {\mathrm{AaDO}}_2 = \left({\mathrm{Patm}\hbox{-}\ \mathrm{P}}_{\mathrm{H}2\mathrm{O}}\right) \times {\mathrm{FiO}}_2\hbox{-}\ {\mathrm{PaO}}_2\hbox{-}\ {\mathrm{PaCO}}_2/\ \mathrm{R}\mathrm{Q} $$

Patm is the atmospheric pressure, which is usually equal to 760 mmHg at sea level but needs to be adjusted in high altitude. P_H2O_ is the pressure of water vapor in one ATM, which is usually considered to be 47 mmHg. RQ is the respiratory quotient and equal to 1 if the energy source is purely carbohydrate or equal to 0.8 when the nutritional source is a combination of carbohydrate, protein, and lipid. The disadvantage of AaDO_2_ is that it does not take ventilator pressure into account.

### Management

The severity of PPHN can range from mild hypoxemia with minimal respiratory distress to severe hypoxemia and cardio-pulmonary instability that requires intensive care support. Infants with PPHN require supportive care tailored to the degree of hypoxemia and physiologic instability. PPHN is often associated with underlying parenchymal lung disease or systemic illness; therapy should target the underlying disease (such as antibiotics for sepsis).

Some hospital based practices such as cesarean sections, induction of labor at late-preterm or early-term gestation, and the use of anesthetics and analgesics delay pulmonary transition at birth. These infants present with “delayed cardiorespiratory adaptation” must be closely monitored and managed with appropriate respiratory support. Respiratory support to recruit the lungs and provide optimal inflation (while avoiding atelectasis and hyperinflation) reduces PVR and is the key step in management of these infants and reduces the risk of PPHN. Many infants with parenchymal lung disease have elevated pulmonary arterial pressures. Premature therapy with early initiation of an inhaled pulmonary vasodilator such as iNO prior to lung recruitment is not beneficial and optimal lung inflation with adequate PEEP and/or surfactant often eliminates the need for specific pulmonary vasodilator therapy.

Mild cases of PPHN with minimal or no respiratory distress can be detected in the newborn nursery either following a desaturation episode or by low postductal oxygen saturation detected on critical congenital heart disease screening [64]. These infants can be managed with supportive care and oxygen supplementation. Close monitoring is important as some of these infants may rapidly deteriorate and require non-invasive ventilation or intubation and mechanical ventilation. Infection should be considered as elevated pulmonary pressures (often associated with systemic hypotension) can be the presenting clinical feature of pneumonia or sepsis.

*Supportive care:* It is important to maintain normothermia and correct metabolic and hematologic abnormalities such as hypoglycemia, hypocalcaemia, acidosis and polycythemia. Sedation may be necessary to provide comfort and decrease oxygen consumption from agitation in hypoxemic or ventilated patients. Paralysis should be avoided if possible, as it has been associated with increased mortality [1]. The goal of medical management is to selectively reduce pulmonary arterial pressure and to maintain systemic blood pressure.

Hyperventilation and alkali infusions to maintain an alkaline pH were strategies previously in use but should be avoided because of associated concerns of impaired cerebral perfusion and sensorineural deafness with respiratory alkalosis have been raised [65,66]. Similar or improved outcomes with less chronic lung disease were also observed in infants with PPHN maintaining normal Pco_2_ (45–60 mmHg) [67,68]. Alkali infusion was associated with increased use of ECMO and need for oxygen at 28 days [1]. Thus, lack of convincing data to support hyperventilation/alkali infusion therapy along with better therapeutic options including inhaled vasodilators have led to decreased use of alkalosis. Most centers avoid acidosis based on animal studies demonstrating exaggerated hypoxic pulmonary vasoconstriction with pH < 7.25 [69]. We recommend maintaining pH > 7.25, preferably 7.30 to 7.40 during the acute phase of PPHN.

### Mechanical ventilation

Given the important contribution of parenchymal lung disease in many cases of PPHN, pharmacologic pulmonary vasodilation alone without lung recruitment would not be expected to cause sustained clinical improvement [70,71]. “Gentle” ventilation strategies with optimal PEEP, relatively low PIP or tidal volume and a degree of permissive hypercapnia are recommended to ensure adequate lung expansion while limiting barotrauma and volutrauma [68,72]. In newborns with severe lung disease, high frequency ventilation is frequently used to optimize lung inflation and minimize lung injury [73]. If a PIP of > 28 cmH_2_O or tidal volumes > 6 cc/kg are required to maintain PaCO_2_ < 60 mmHg on conventional ventilation, we recommend switching to high frequency (jet or oscillator) ventilation. In clinical studies using iNO, the combination of high frequency ventilation and iNO resulted in the greatest improvement in oxygenation in PPHN associated with diffuse parenchymal lung disease such as RDS and pneumonia [74,75].

Oxygen is a specific and potent pulmonary vasodilator and increased oxygen tension is an important mediator of reduction in PVR at birth. Avoiding hypoxemia by mechanical ventilation with high concentrations of oxygen used to be a mainstay of PPHN management. Fetal lamb studies demonstrate that increased fetal oxygen tension augments endogenous NO release [76] and increased pulmonary blood flow induced by rhythmic distention of the lung and oxygen are mediated in part by endogenous NO [77]. However, it has also been shown that brief exposure to 100% oxygen in newborn lambs results in increased contractility of pulmonary arteries [78], reduces response to iNO [79,80] and increases the potential for oxidative stress [81]. Reactive oxygen species (ROS), such as superoxide interact with NO to form a potent oxidant peroxynitrite capable of vasoconstriction and surfactant inactivation [82]. In addition to direct inactivation of NO, ROS can decrease eNOS activity, sGC activity and increase PDE5 activity, resulting in decreased cGMP levels and potentiation of pulmonary vasoconstriction. In the ovine ductal ligation model of PPHN, maintaining oxygen saturations in the 90-97% range results in low PVR [80]. We recommend maintaining preductal oxygen saturations in low to mid-90s with PaO_2_ levels between 55 and 80 mmHg during management of infants with PPHN.

### Surfactant

Exogenous surfactant therapy improved oxygenation and reduced the need for extracorporeal membrane oxygenation (ECMO) in neonates with PPHN secondary to parenchymal lung disease such as RDS, pneumonia/sepsis or MAS. This multicenter trial also demonstrated that this benefit was greatest for infants with mild to moderate disease, and with an OI of 15–25 [83]. A *post-hoc* analysis of the randomized trial of early nitric oxide use showed that early use of surfactant prior to randomization decreased the risk of death/ECMO especially in infants with parenchymal lung disease [84]. Over the past decade, the use of surfactant in treating secondary PPHN and respiratory failure has increased and might have contributed to improved effectiveness of iNO with reduced need for ECMO. Surfactant inactivation and deficiency are observed in many neonatal respiratory disorders such as pneumonia, RDS and MAS. We recommend that infants with PPHN secondary to parenchymal lung disease receive a dose of surfactant rich in surfactant protein-B (SP-B), such as calfactant - Infasurf® (ONY Inc, Amherst NY) or poractant-α - Curosurf® (Chiesi Farmaceutici, S.p.A, Parma, Italy). Synthetic surfactant (lucinactant, Surfaxin, Discovery laboratories, Inc. Warrington PA) rich in SP-B mimetics has been shown to be resistant to inactivation and effective in animal studies [85,86] and appears to be safe in children with HRF [87] but its efficacy in term infants with hypoxic respiratory failure is not known.

It is not clear if surfactant therapy is beneficial in infants with CDH. Animal studies show benefit [88-90] but a review of the CDH registry did not support the use of surfactant [91]. We recommend administration of surfactant only in the presence of clinical, radiological or biochemical evidence of surfactant deficiency in CDH and administer only 50% of the dose because of pulmonary hypoplasia.

### Inhaled Nitric Oxide (iNO)

Nitric Oxide is a potent vasodilator that has also been shown to be an important regulator of vascular tone, growth and remodeling [92]. In the endothelium, NO is produced from the terminal guanidino nitrogen of L-arginine on its conversion to L-citrulline by the enzyme eNOS in a reaction that requires molecular oxygen [92].

As an inhaled vasodilator, iNO selectively dilates the pulmonary circulation without a significant decrease in systemic blood pressure (*selective effect of iNO).* Inhaled NO is also preferentially distributed to the ventilated segments of the lung, resulting in increased perfusion of the ventilated segments, optimizing VQ match (*micro-selective effect of iNO)*. Studies have shown that iNO therapy causes marked improvement in oxygenation in term newborns with PPHN [93]. Multicenter randomized clinical studies subsequently confirmed that iNO therapy reduces the need for ECMO in term neonates with hypoxemic respiratory failure [94-96]. Inhaled NO therapy has been approved by the FDA for clinical use in term/near term newborn infants (>34 wks gestation) with hypoxic respiratory failure and PPHN since 2000.

*Initiation of iNO*: There has been a debate regarding the timing of initiation and optimum starting dose of iNO in PPHN. An OI of 25 is associated with a 50% risk of requiring ECMO or mortality [97] in the absence of specific pulmonary vasodilator therapy. Konduri et al. initially demonstrated that earlier initiation of iNO with an OI of 15–25 did not reduce the need for ECMO but may have a tendency to reduce the risk of progression to severe hypoxemic respiratory failure [98]. *Post-hoc* analysis of the same study suggested that the use of surfactant prior to randomization and enrollment (and use of iNO) at an OI of ≤ 20 was associated with reduced incidence of ECMO/death [84].

**Figure 4:**
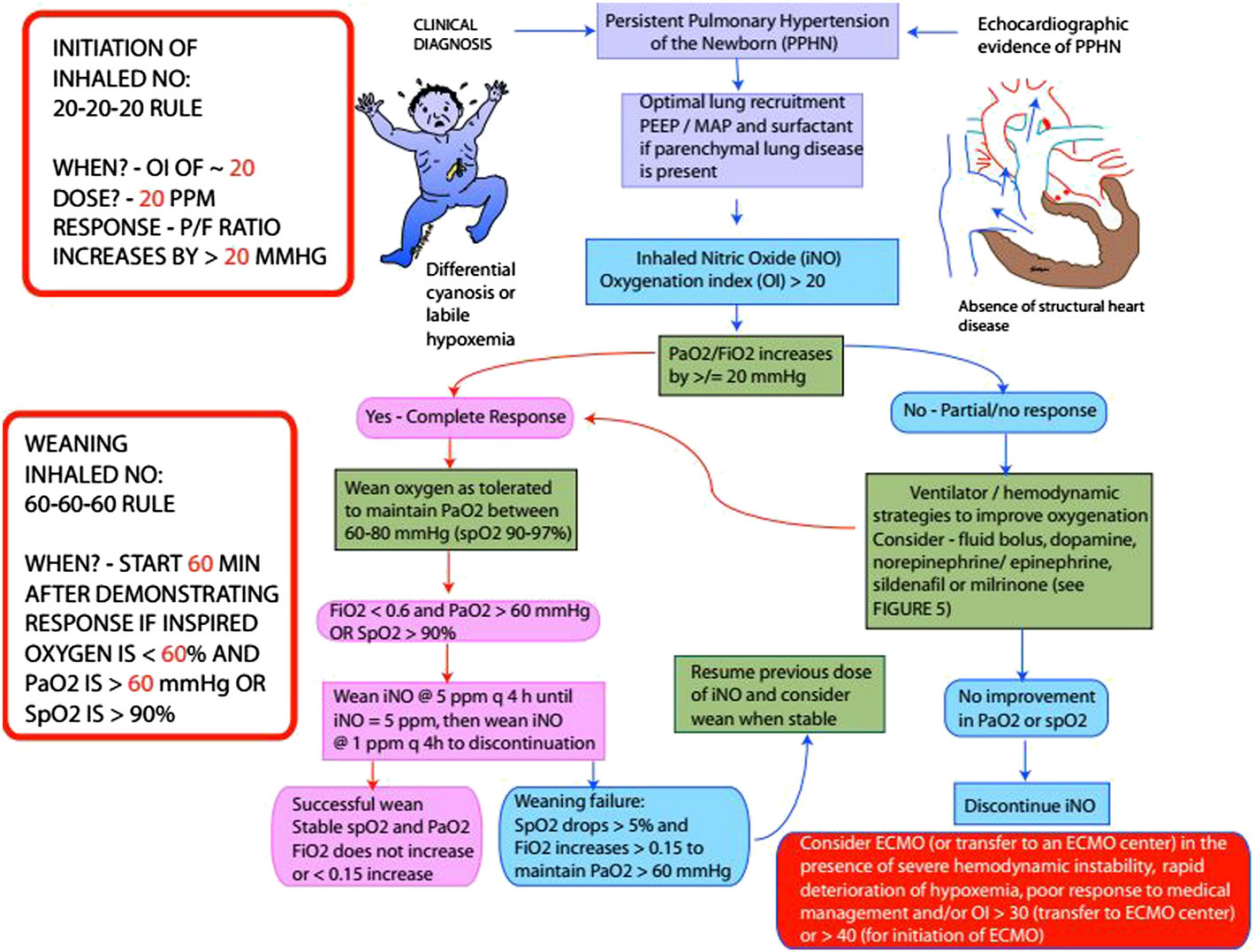
Inhaled Nitric Oxide therapy in neonates with PPHN: The diagnosis of PPHN is based on clinical (labile hypoxemia and differential cyanosis) or echocardiographic findings. Optimal oxygenation and lung recruitment with optimal lung inflation and surfactant in the presence of parenchymal lung disease is the initial step in the management of PPHN. An OI of 20 is a reasonable threshold to initiate iNO. The presence of hemodynamic instability and severe hypoxemia (OI ~ 40 range) is an indication for ECMO. See text for details. *(copyright Satyan Lakshminrusimha)*.

*Dosing of iNO:* Previous clinical trials suggested that the ideal starting dose for iNO is 20 parts per million (ppm) with the effective doses between 5 and 20 ppm [99]. Doses > 20 ppm did not increase the efficacy and were associated with more adverse effects in these infants [95] such as elevated methemoglobin (>7%) and nitrogen dioxide (NO_2_) (>3 ppm) [93]. A dose of 5 ppm results in improved oxygenation in PPHN. A dose of 20 ppm results in improved oxygenation *and* results in the most optimal decrease in pulmonary to systemic arterial pressure ratio [100]. To summarize, we recommend initiation of iNO if OI is ~ **20** at a dose of **20** ppm. A complete response to iNO is defined as an increase in PaO_2_/ FiO_2_ ratio of ≥ **20** mmHg. (20-20-20 rule for initiation of iNO, Figure 4). Methemoglobin levels are monitored at 2 and 8 hours after initiation of iNO and then once a day for the duration of iNO therapy. Some centers stop checking methemoglobin levels after the first couple of days if levels are low (<2%) and iNO dose remains ≤ 20 ppm. High inspired oxygen and high mean iNO dose are risk factors for elevated methemoglobin in term infants [101].

*Weaning iNO*: Due to rebound vasoconstriction and resultant pulmonary hypertension on abrupt withdrawal, iNO needs to be weaned gradually [102]. Weaning in steps from 20 ppm gradually over a period of time before its discontinuation has been shown to prevent the rebound effect [103]. If there is oxygenation response, inspired oxygen concentration is first weaned below **60**% and then iNO is weaned only if PaO_2_ can be maintained ≥ **60** mmHg (or preductal SpO_2_ ≥ 90%) for **60** min (60-60-60 rule of weaning iNO). At our center, we wean iNO at a rate of 5 ppm every 4 hours. Once iNO dose is 5 ppm, gradual weaning by 1 ppm q 4 hours is performed (Figure 4). Continuing iNO in the absence of a response or not weaning iNO or extremely slow weaning can potentially lead to suppression of endogenous eNOS [104,105].

### Management of iNO-resistant PPHN

**Figure 5:**
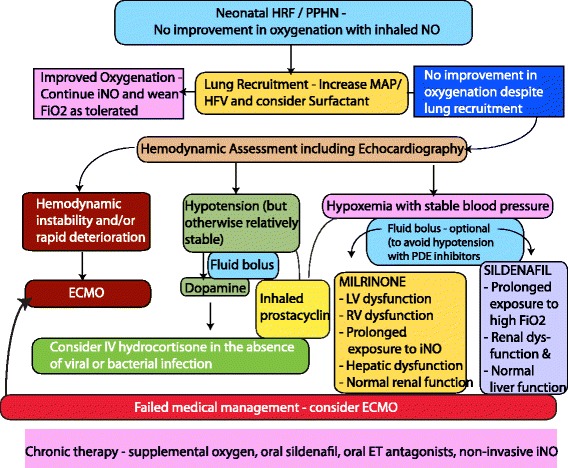
Flow chart showing the author’s suggested guidelines for management of iNO resistant PPHN. *(copyright Satyan Lakshminrusimha)*.

In approximately a third of term and near-term infants with PPHN, iNO does not result in sustained improvement in oxygenation [95]. Adequate lung recruitment (with surfactant and/or optimal PEEP/MAP preferably with high frequency ventilation) is crucial to deliver iNO to its target site – the pulmonary vasculature [106]. A repeat echocardiogram to evaluate ventricular function and severity of PPHN (and to rule out cyanotic CHD such as total anomalous pulmonary venous return (TAPVR) that may have been missed on the first echocardiogram [107]) is the next step. Management of systemic hypotension in PPHN is discussed below. If lung recruitment and hemodynamic stability are achieved and iNO is still not effective, patient should be managed in a tertiary center with access to ECMO. Our recommendations for management of iNO-resistant PPHN not responding to iNO in spite of lung recruitment with increased MAP and surfactant are outlined in Figure 5 and summarized here.

1. Hemodynamic evaluation: A repeat echocardiogram should be performed to evaluate structural heart disease, left ventricular dysfunction, right ventricular dysfunction, and ventricular output For example, if left ventricular dysfunction is associated high left atrial pressures and a left-to-right shunt at the level of the oval foramen in the presence of a right-to-left shunt at the ductus arteriosus, iNO is contraindicated and an inodilator such as milrinone should be initiated.
2. Rapid deterioration with hemodynamic instability should necessitate cannulation for ECMO (or immediate transfer to an ECMO center).
3. In the presence of systemic hypotension, a fluid bolus (10 ml/kg of Lactated Ringers or isotonic saline) followed by dopamine is recommended. Some centers prefer the use of norepinephrine or vasopressin. A cortisol level is drawn in these patients. If the levels are low relative to the infant’s stress level and there is no evidence of infection (viral or bacterial), the authors recommend a stress dose of hydrocortisone.
4. If blood pressure is relatively stable but hypoxemia persists, consider the use of PDE inhibitors. Sildenafil is preferred if normal liver and ventricular function are present and may have added benefit in the context of prolonged hyperoxia. Ventricular dysfunction or hepatic compromise are indications for Milrinone rather than sildenafil as long as normal renal function is present. Chronic therapy (especially in the presence of CDH or BPD) involves PDE 5 inhibitors and endothelin receptor antagonists and non-invasive iNO (Figure 5).

### Prostaglandin E1 (PGE1)

Aerosolized prostaglandin E1 (Alprostadil) has been used to treat pulmonary hypertension in adults. In a small pilot phase I-II study, Sood et al. suggested that inhaled PGE1 was a safe and selective pulmonary vasodilator in hypoxemic respiratory failure with or without use of iNO [108]. PGE1 solution for aerosolization is prepared from Alprostadil® (Prostin VR 500, Pfizer, New York NY) and administered as a continuous nebulization through a MiniHeart low flow jet nebulizer (WestMed Inc, Tuczon, AZ) at 150–300 ng/kg/min diluted in saline to provide 4 ml/hr [109].

Intravenous PGE1 has also been used in patients with CDH in combination with iNO to promote pulmonary vasodilation and to maintain ductal patency and reduce right ventricular afterload [110].

### Inhaled Prostacyclin (PGI_2_)

Prostacyclin administered intravenously is a common therapy in adults with pulmonary arterial hypertension. Inhaled PGI_2_ has been used in PPHN resistant to iNO at a dose of 50 ng/kg/min. [111] The intravenous formulation Flolan° (Glaxo-Wellcome, Middlesex, UK) is dissolved in 20 ml of manufacturer’s diluent (a glycine buffer, pH −10). Fresh solution is added to the nebulization chamber every 4 hours [111]. The effect of such alkaline pH on neonatal respiratory tract is not known.

### Phosphodiesterase inhibitors

*a) Sildenafil* (PDE 5 Inhibitor): Studies have shown that cGMP is decreased in response to exogenous NO in animal models of PPHN [112], and increased clearance of cGMP by one or more phosphodiesterases has been proposed as one of the causes [113]. Sildenafil acts by inhibiting cGMP-specific phosphodiesterase type 5 (PDE 5), an enzyme that promotes degradation of cGMP. Studies have shown that oral sildenafil (dose range 1–2 mg/kg every 6 h) improves oxygenation and reduces mortality, in centers limited by non-availability of iNO and ECMO [114,115].

Intravenous sildenafil was shown to be effective in improving oxygenation in patients with PPHN with and without prior exposure to iNO [116]. The use of intravenous sildenafil should be restricted to refractory cases at a center with ECMO back-up, due the potential risk of systemic hypotension [117] and pulmonary hemorrhage, presumably due to sudden reversal of ductal shunt [118]. Based on pharmacokinetic data in neonates with PPHN, intravenous sildenafil is administered as a load of 0.42 mg/kg over 3 hours (0.14 mg/kg/h) followed by 1.6 mg/kg/day as a continuous maintenance infusion (0.07 mg/kg/h).

*b) Milrinone* (PDE 3 Inhibitor): Milrinone inhibits PDE3 and increases concentration of cAMP in pulmonary and systemic arterial smooth muscle and in cardiac muscle. Milrinone relaxes pulmonary arteries in the fetal lamb model of PPHN [119]. Infants with PPHN refractory to iNO therapy have responded to IV milrinone in 3 case series [120-122] and appears to be particularly useful in the presence of ventricular dysfunction [123,124]. A loading dose (50 mcg/kg over 30–60 min) followed by a maintenance dose (0.33 mcg/kg/min and escalated to 0.66 and then to 1 mcg/kg/min based on response) is commonly used. The loading dose is not recommended in the presence of systemic hypotension and in premature neonates [123]. As with any systemic vasodilator, hypotension is a clinical concern and blood pressure needs to be closely monitored. A fluid bolus (10 ml/kg of Lactated Ringer’s solution) prior to loading dose may decrease the risk of hypotension. In addition, one case series described an increased incidence of intracranial hemorrhage with the use of milrinone in PPHN [121]. Milrinone may be the pulmonary vasodilator of choice in the presence of PPHN with left ventricular dysfunction (Figure 5).

### Bosentan (Endothelin-1 receptor blocker)

Endothelin receptor antagonists are beneficial and well tolerated in adult patients with pulmonary arterial hypertension [125]. Initial reports suggested that bosentan was an effective drug in the management of PPHN [126]. The results of a multi-center, randomized, double-blind, placebo-controlled exploratory trial of bosentan in PPHN was recently reported. Bosentan (2 mg/kg/dose BID) did not show any additive effect on top of iNO in term neonates with PPHN [127]. However, endothelin receptor antagonists may have a role in the management of chronic pulmonary hypertension associated with BPD or CDH.

### Steroids

Antenatal betamethasone attenuated oxidative stress and improved in vitro response to vasodilators in a fetal lamb model of pulmonary hypertension [128]. Glucocorticoids have been found to improve oxygenation and attenuate the pulmonary hypertensive response in animal models of meconium aspiration syndrome, which is a common cause of PPHN [129]. Steroids have been reported to decrease hospital stay and duration of oxygen use in infants with meconium aspiration [130,131]. It is proposed that hydrocortisone attenuates ROS production by induction of superoxide dismutase and normalization of PDE5 activity [132]. Looking at the evidence this far, we do not recommend routine use of steroids in patients with PPHN especially if there is suspicion of viral or bacterial sepsis. Anecdotal use of stress dose hydrocortisone in iNO resistant PPHN associated with systemic hypotension in our unit has resulted in stabilization of systemic blood pressure and improved oxygenation possibly secondary to hemodynamic stability and PDE-5 inhibitory effects [133].

### Extracorporeal Membrane Oxygenation (ECMO)

ECMO is a technique of modified cardiopulmonary bypass used over a prolonged period to support heart and lung function. The use of neonatal ECMO has declined from a peak of 1516 cases per year in 1992 to 750–865 cases/year from 2008 to 2012. This decline is likely due to improvements in both perinatal care and availability of advanced therapies for neonatal hypoxemic respiratory failure including high-frequency ventilators, surfactant, and iNO [134]. In newborns with PPHN, mechanical ventilation with oxygen and iNO is the initial treatment, but prolongation of iNO with high oxygen levels may induce chronic lung disease and extend the length of stay in the NICU [135]. On the other hand, initiating ECMO too early may expose newborns to major vessel cannulation and systemic anticoagulation [136]. General accepted criteria to start ECMO is persistent hypoxemia (with an OI of >40 or AaDO2 > 600 in spite of aggressive medical management of PPHN with mechanical ventilation and iNO) and the presence of hemodynamic instability (Figure 5).

### Management of systemic hypotension in PPHN

**Figure 6:**
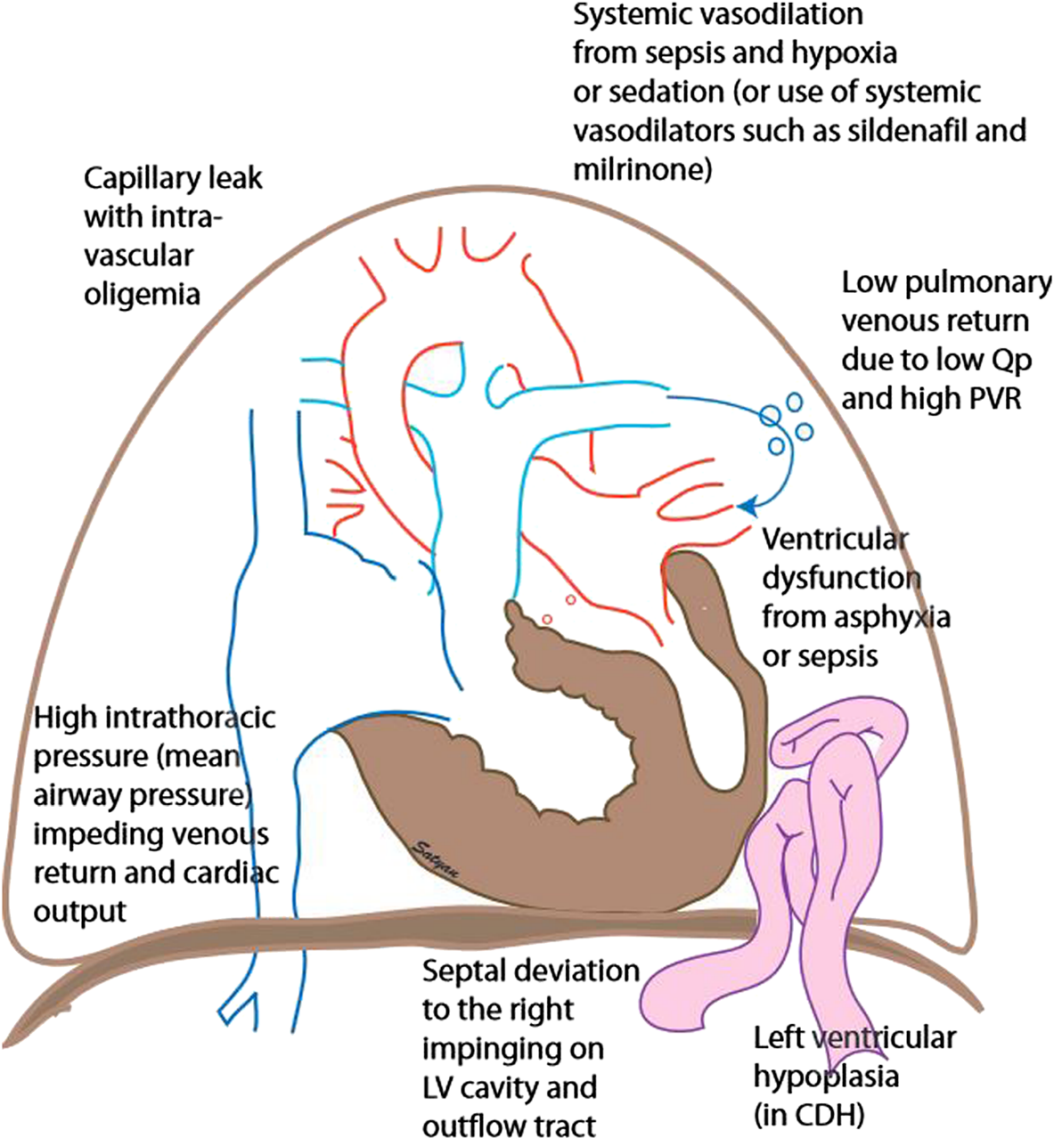
Causes of hypotension in infants with PPHN: Sepsis and hypoxia causes intravascular oligemia due to capillary leak or due to systemic vasodilation. Neonates with PPHN often require high mean airway pressure that impedes venous return to the right side of heart. Increased pulmonary vascular resistance results in less blood flow through pulmonary circulation reducing left ventricular preload. The deviation of the interventricular septum towards left ventricle further impedes left ventricular filling. Neonates with PPHN secondary to sepsis, hypoxia and CDH can also experience systemic hypotension secondary to left ventricular dysfunction. *(copyright Satyan Lakshminrusimha)*.

Systemic hypotension is common in infants with PPHN and the causes are outlined in Figure 6. Decreased systemic blood pressure exacerbates right-to-left shunt and worsens hypoxemia in PPHN. The cause of systemic hypotension should be addressed first – administration of volume bolus in hypovolemia, decrease in MAP in the presence of hyperinflation and antibiotics for sepsis. The use of dopamine to increase systemic blood pressure to reduce right-to-left shunt is a common practice. Such a practice is effective in the presence of systemic hypotension. However, increasing systemic pressure to supraphysiologic levels is not recommended. In some patients with PPHN, the presence of a patent ductus arteriosus acts as a pop-off valve, limiting right ventricular preload and dysfunction. Increasing systemic blood pressure limits right-to-left shunt across the PDA and may add to right ventricular strain. In addition, the optimal therapy for reduced pulmonary blood flow is selective pulmonary vasodilation. Instead, if pulmonary blood is forced by higher systemic pressure (by limiting right-to-left shunts) through a constricted pulmonary circuit, endothelial dysfunction due to increased shear stress [47] is likely to exacerbate PPHN. Dopamine (especially at > 10 mcg/kg/min) is not selective to systemic vasculature and can increase pulmonary arterial pressure in PPHN [106]. Norepinephrine infusion is also effective in stabilizing systemic blood pressure and improving oxygenation in neonates with PPHN [137]. Vasopressin may be an effective therapeutic agent with some selectivity to systemic vasoconstriction [138]. As mentioned in the previous paragraph, hydrocortisone may also be effective to stabilize blood pressure in PPHN.

### Asphyxia, hypothermia and management of PPHN

**Figure 7:**
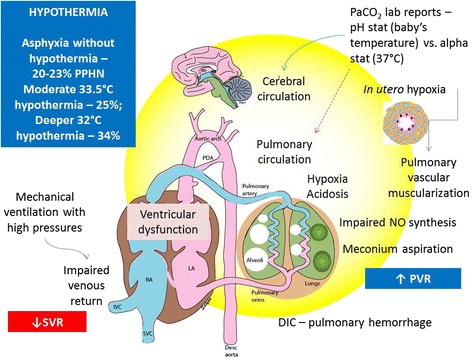
Asphyxia and PPHN: Fetal hypoxia (secondary to *in-utero* asphyxia and meconium aspiration) causes pulmonary vascular remodeling, which down regulates iNO signaling pathways and causes PPHN. In infants with perinatal hypoxia, the combination of hypoxia and acidosis increases the risk of PPHN. Preexisting PPHN may be exacerbated by therapeutic hypothermia. Errors in PaCO_2_ measurement secondary to body temperature changes may result in fluctuations in PCO_2_ leading to changes in cerebral and pulmonary vascular resistance (see text for details). *(copyright Satyan Lakshminrusimha)*.

Asphyxia is associated with hypoxemia and acidosis. Infants with asphyxia also have evidence of surfactant deficiency and/or meconium aspiration syndrome (Figure 7) [139]. The use of moderate hypothermia (33.5°C for 72 hours) does not result in a significant increase in the incidence of PPHN (25% vs. 22% with conventional management without hypothermia) [140]. However, as compared to moderated hypothermia (33.5°C), deeper whole-body cooling to 32°C is associated with a tendency to increased PPHN (34 vs 25%, p-0.06), increased need for inhaled NO (34 vs 24%, p-0.03) and ECMO (9 vs 4%, p-0.005) [141]. Case reports indicate that patients with hypoxemic respiratory disorders prior to the onset of cooling (especially those that need > 50% inspired oxygen and/or iNO) [142], may experience exacerbation of PPHN with hypothermia and/or rewarming [143]. Mild therapeutic hypothermia by itself is not a cause for PPHN. However, infants predisposed to elevated PVR due to the presence of asphyxia and respiratory disease may not tolerate hypothermia induced pulmonary vasoconstriction [144]. These findings emphasize the need for close monitoring of core temperature, systemic/pulmonary hemodynamics and oxygenation during hypothermia and rewarming for asphyxia.

In many centers, confusion exists regarding optimal reporting of PaCO_2_ during whole-body hypothermia. The laboratory may report PaCO_2_ levels either at baby’s temperature (known as the pH-stat method) or corrected for 37°C (alpha-stat method). Decreasing temperature increases the solubility of CO_2_ in the blood and decreases PaCO_2_ and may have implications for PPHN management with potential of overventilation or underventilation. We recommend the pH-stat method and reporting of PaCO_2_ at actual (and not corrected) body temperature.

### Long term outcome of PPHN

PPHN is a syndrome associated with significant long-term morbidity, irrespective of the treatment modality. These infants suffer from long-term consequences such as neurodevelopmental, cognitive and hearing abnormalities [145-147]. Thus, it is essential to provide long-term multidisciplinary follow-up after discharge. Konduri et al. in their long-term follow-up of infants randomized to early iNO in PPHN, noted neurodevelopmental impairment in about 25% of infants and hearing impairment in approximately 23% [145]. Long-term neurodevelopmental outcome at school age for neonates with PPHN critical enough to receive inhaled NO or ECMO is generally encouraging. Rosenberg et al. reported that among 109 school age survivors of PPHN (77 of whom received inhaled NO and 12 that required ECMO), medical, neurodevelopmental and behavioral outcomes did not differ between children treated with iNO, with or without ECMO, and those managed with no exposure to iNO. However, 24% had persistent respiratory problems, 60% had abnormal chest X-rays and 6.4% had some degree of sensorineural hearing loss. Overall, 9.2% of the cohort had a full scale IQ less than 70 and 7.4% had an IQ from 70 to 84 [148]. The UK collaborative trial randomized critically ill neonates into transfer to a regional center for ECMO or continued conventional care at the local NICU. At 7 year follow-up, mortality was significantly lower in the ECMO group with no increase in disability [149]. The presence of neurodevelopmental and medical disabilities may reflect the severity of the underlying illnesses experienced by these infants rather than complications of iNO or ECMO. However, therapeutic interventions such as hyperventilation are associated with sensorineural hearing loss and must be avoided [66,150].

## Conclusions

Over the last two decades, management of infants with PPHN has included improved with improved ventilation strategies to optimize lung recruitment, provide “gentle” ventilation and minimize oxygen toxicity paired with the therapeutic use of surfactant and iNO. These changes have led to a substantial decrease in the number of neonatal PPHN patients requiring ECMO for respiratory disorders. Animal models have contributed to our understanding of fetal circulation, pulmonary vascular transition at birth and hemodynamic and biochemical abnormalities associate with PPHN. Further clinical research into pulmonary vasodilator therapy, reversal of remodeling of the pulmonary vasculature and right ventricle are crucial. Two challenges which remain in the field of PPHN include management of pulmonary hypoplasia and pulmonary hypertension in CDH and BPD-associated pulmonary hypertension in the premature infant [97]. In addition, asphyxia (with or without MAS and/or therapeutic hypothermia) remains an important cause for PPHN worldwide. Further research to evaluate and develop appropriate strategies to ameliorate pulmonary vascular disease in these conditions are warranted.

## Abbreviations

AaDO2: Alveolar-arterial oxygen difference
ACD: Alveolar Capillary Dysplasia
ANP: Atrial Natriuretic Peptide
ATP: Adenosine Triphosphate
BNP: B-type Natriuretic Peptide
BPD: Broncho Pulmonary Dysplasia
CHD: Congenital Heart Disease
CGMP: Cyclic guanosine monophosphate
CNP: C-type Natriuretic Peptide
ECMO: Extracorporeal Membrane Oxygenation
ENOS: Endothelial Nitric oxide synthases
FDA: Food and Drug Administration
FiO2: Fraction of Inspired Oxygen
iNO: Inhaled Nitric Oxide
MAP: Mean Airway Pressure
MAS: Meconium Aspiration Syndrome
NO: Nitric Oxide
NOS: Nitric Oxide Synthase
NSAIDS: Non-Steroidal Anti-Inflammatory Drugs
OI: Oxygenation Index
PDA: Patent Ductus Arteriosus
PDE: Phosphodiesterase
PEEP: Peak End Expiratory Pressure
PFO: Patent Foramen Ovale
PI: Pulsatility Index
PIP: Peak Inspiratory Pressures
PPHN: Persistent Pulmonary Hypertension
PVR: Pulmonary Vascular Resistance
RDS: Respiratory Distress Syndrome
ROS: Reactive Oxygen Species
RQ: Respiratory Quotient
sGC: Soluble Guanylate Cyclase
SP-B: Surfactant Protein-B
SSRI: Selective Serotonin Reuptake Inhibitors
SVR: Systemic Vascular Resistance
VQ: Ventilation-perfusion
